# The use of Online Consumer reviews in the Surveillance and Detection of Infectious Gastrointestinal Disease Outbreaks

**DOI:** 10.23889/ijpds.v11i4.3808

**Published:** 2026-07-06

**Authors:** Ankit Gupta, Priti Sheoran, Rakesh Chandra

**Affiliations:** 1 International Institute for Population Sciences, Mumbai, India; 2 Indira Gandhi National Tribal University, Amarkantak, India; 3 Tata Institute for Social Sciences, Mumbai, India

## Background

Antenatal care is a critical component of maternal health, aiming to reduce pregnancy-related risks through timely check-ups and early detection of complications. Despite improvements in India’s maternal healthcare coverage, substantial disparities exist across geographic regions, particularly between hilly and non-hilly areas. Understanding the determinants of ANC 4+ utilization in such settings is essential for targeted interventions.

## Objectives

1. The overall prevalence of ANC 4+ utilization in Hilly and Non-Hilly states in India
2. Influence of predisposing, enabling, and need factors—guided by Andersen’s Behavioral Model on ANC 4+ uptake across hilly and non-hilly regions using NFHS-5 State Module data.

## Data and Methods

Data were drawn from the NFHS-5 State Module, comprising 26,983 households and 15,935 women who received at least four ANC visits and 11,048 women who received fewer than four visits. The dependent variable, ANC 4+, was dichotomized (≥4 visits vs <4). Predictor variables were classified into predisposing, enabling, and need factors following Andersen’s framework. Weighted descriptive statistics, bivariate associations, multivariable logistic regression were used to identify significant determinants.

## Results

Preliminary analysis shows notable geographic contrasts: women residing in non-hilly regions had higher ANC 4+ utilization compared to those in hilly areas. Predisposing factors such as maternal age, caste, religion, mass media exposure, and birth order displayed significant associations with ANC coverage. Enabling factors, including wealth index, health insurance, place of delivery, and distance to health facility, were strongly predictive of ANC 4+ uptake. Need factors such as the presence of non-communicable diseases (NCDs) also influenced utilization patterns.

